# The role of gender in providing expert advice on cyber conflict and artificial intelligence for military personnel

**DOI:** 10.3389/fdata.2022.992620

**Published:** 2022-09-30

**Authors:** Kelly Fisher

**Affiliations:** Department of Social Dynamics, Peace Research Institute Oslo, Oslo, Norway

**Keywords:** AI, gender, cyber, military, communication, masculinity

## Abstract

This article draws upon original qualitative interview data with Norwegian male and female cyberengineer cadets at the Norwegian Cyber Defense Academy, who could in the future be working with AI-enabled systems in a variety of positions throughout the Norwegian military. The interviews explored how these cadets feel they as cyberengineers will be perceived in their future positions in the military, what challenges they feel they may face, and how gender may play a role in this. Different cyberengineers expressed concern about being able to communicate the cyber domain to their non-technology specialist colleagues due to the increasing complexity of new technologies. Gender appeared to be playing a role in this concern as the women interviewed expressed specific concerns that they feel as women, that they do not fit the stereotype of who is a cyberengineer, while some of the men felt that as cyberengineers they were seen as embodying a nerd masculinity, and that these gendered perceptions has implications for how they feel others perceive their competence levels. The findings from this article highlights gendered hierarchies in the military and the need for military institutions to focus on developing communication skills among those working with cyber operations. As the role of cyber is expected to grow in military operations, cyberengineers will need to find ways of communicating effectively with non-specialists—especially as complex AI-enabled systems are introduced. Finally, this paper argues the need for military institutions to take gender into account for this training and need for gender-sensitive policies.

## Introduction

The rapid development of new technologies in society is resulting in many changes in how warfare is conducted (Feickert, 2021). One of those changes is the increasingly large role that cyber operations play, whether during actual warfare or in gray-zone conflict (Bilal, 2021). Due to the broad scope of what is cyber operations, definitional clarity is challenging and with no generally agreed upon definition of what cyber operations are (Dinstein and Dahl, 2020), or the cyber domain for that matter (Ringas et al., 2014). For the purpose of this paper, cyber operations are defined as an attack by an actor (nation, non-state actors) upon another's cyber capabilities (Dinstein and Dahl, 2020), with the cyber domain being defined as including computers, networks, and anything else connected to the internet and communication capabilities (Ringas et al., 2014, p. 58). While still in its infancy, Artificial Intelligence (AI) is already playing a role in cyber operations, and this is expected to grow in the years ahead, both for offensive and defensive purposes (National Security Agency, 2021; Helkala et al., 2022).

A new challenge emerging from the increased complexity of these technologies is that troops and commanding officers and non-technology specialists in the military often have minimal understanding of the cyber domain (Jøsok et al., 2017). As a result, cyber operators have a greater responsibility to communicate effectively what is happening in the cyber domain to their commanding officers and fellow troops (Knox et al., 2018), especially as they will need to work closely during “multi-domain operations” (Feickert, 2021). A growing field of research has highlighted the importance of good communication skills for cyber professionals, and the ability to explain ongoings in cyber domain to their less tech-savvy colleagues (Dawson and Thomson, 2018). Furthermore, this poses questions of how the implementation of AI-enabled systems will contribute to the challenge of communicating complex technologies, as AI further obscures understanding how these technologies work (Ellis and Grzegorzewski, 2021).

Possible uses of AI in cyber operations may include programs that detect and then respond to malicious activity in the military's networks, and at a rate faster than humans could (Helkala et al., 2022). While there is enthusiasm for the use of AI, there are also concerns about unintended consequences resulting from its use (Ellis and Grzegorzewski, 2021). As many at the top of the command hierarchy would be held accountable for unintended consequences of AI use, there is likely to be hesitancy about deploying these AI-enabled systems (Helkala et al., 2022). Concerns about unintended consequences from AI exists across a number of different sectors, including both military and non-military (Steen et al., 2021). As these types of systems can offer important advantages in cyber operations, cyberengineers will need to be able to explain these systems in a way that ranking officers can understand.

However, it is not only an issue that cyber professionals need to be able explain these technologies to their colleagues, but also there is the matter of how these cyber professionals are viewed and perceived by their colleagues. Expertise and those seen as experts is relational (Collins and Evans, 2007), meaning that it is also a matter of how individuals are perceived regarding their level of expertise. Many factors can play a role in how someone is perceived, including gender (Ore, 2018). This raises a question of how gender might play a role in how cyber specialists feel they are perceived. Research has shown the gender biases that exist against female experts in a number of fields (Greve-Poulsen et al., 2021, p. 2), including in cybersecurity (Frieze and Quesenberry, 2019). While there is research outlining the importance of good communication skills amongst those working in cyber operations, including in the Norwegian military's Cyber Defense (Knox et al., 2018), few of these have taken into account how gender may play a role in this (Ask et al., 2021; pp. 33–35).

To address this knowledge gap, original qualitative interviews were carried out with cadets at the Norwegian Defense Cyber Academy. These cadets are in the final year of their education and will be deployed throughout the Norwegian military to support cyber capabilities. The cadets and types of tasks they will be working with are those in which AI-enabled programs may soon come to play a role, providing an opportunity to understand what challenges may exist for cyber cadets and how military training and educational institutions can try to address this issue. The main questions explored in this article are how these cadets feel they as cyberengineers will be perceived in their future positions in the military, what challenges they feel they may face as cyberengineers, and how gender may play a role in this. The implications of exploring perceptions of current students allows for institutions to explore how these perceptions align or differ from future working situations, and then aim to better prepare their students for future realities trainings and curriculum (Sipe et al., 2010; p. 345).

Norway's military presents an interesting case as its military is often praised for its efforts of having a gender balanced and inclusive military. Since 2015 Norway has had universal conscription for both men and women (Jakobsen, 2021) and in 2020 19% of Norwegian military personnel were women with 33% of all conscripts being women (Forsvaret, 2021). Despite this, different studies have been carried out showing the way in which women still face barriers to inclusion in the Norwegian military (Kvarving, 2019). However, little of this research has focused on female cyber cadets in the Norwegian military. Drawing from research findings on women working in the cybersecurity industry and IT field more broadly globally (Frieze and Quesenberry, 2019) and in Norway (Corneliussen, 2021), we can see that women face gendered stereotypes of who is seen as being technically competent. As Corneliussen (2021) found in research on women working in ICT, most of these women perceive and experience that technology is something seen as masculine, and an environment in which they face different barriers to inclusion. The findings from these interviews aim to contribute further knowledge both to gender dynamics in the military (Enloe, 1989) as well as those working with technology (Wajcman, 2000), and in the specific case of Norwegian cyberengineers, where those two fields overlap. Finally, this article highlights the relevance and importance of gender in understanding not only women's experiences, but men's experiences in the military (Christensen and Kyed, 2022), and aims to build upon a growing field of literature examining how new and emerging technologies are disrupting and reenforcing gendered hierarchies in the military (Clark, 2018).

In the next section the methods carried out for this project are described. Following this the results based upon the qualitative interviews are presented. The final section is a discussion, and the paper concludes with recommendations for future research.

## Methods

This paper is based upon semi-structured interviews with cadets at the Norwegian Defense Cyber Academy. Thirteen cadets were interviewed who are in the final year of their bachelor's degree in cyberengineering, which is about 1/3 of the class. The cyberengineering program is a combination bachelor degree where students receive training in telematics, cyberengineering, and military leadership. Ten of the cadets were men, and three were women. This represents a similar gender ratio of cadets studying cyberengineering at the Cyber Academy, where each class has about 50 students, and where usually between 15 and 30% of each cohort in recent years has been women.

Cyberengineering cadets generally are deployed across the whole military and may work in a number of roles, from maintaining radios and communications for field units, to working at the main office for the Norwegian Cyber Defence Force in Lillehammer. Cadets were chosen as AI-enabled systems use in the Norwegian military currently is limited or non-existent, and these cadets will likely be working with such systems or overseeing others using them in their future military career. Speaking with cadets rather than currently deployed cyberengineers presents opportunities to explore how they perceive their future roles, which can provide insights for training institutions on how they can better prepare their cadets for the realities of the field, which may differ from their perceptions (Sipe et al., 2010).

This study was approved by the Defense Force, and participation was completely voluntary. Cadets were sent initial information about the project and participation *via* email through their course instructor, and interested cadets then emailed back. All cadets received a consent form and were informed of their rights in line with the regulations carried out by the Norwegian Centre for Research Data (NSD).

Semi-structured interviews were carried out as they allowed me to maintain some order in the interview, while also being able to explore themes that emerged during the interview (Morris, 2015). Semi-structured interviews also enable a more conversational dynamic, where the interviewer asks questions but where the participants are able to express themselves as they desire (Morris, 2015, p. 3). As someone whose Norwegian competence is only moderate the interviews were carried out in English, which raises important questions about possible language-related challenges. My interview guide was designed with this under consideration, and while most cadets were comfortable speaking in English, the cadets were given the choice to speak in Norwegian if ever they were uncertain of how to express something.

As the scope of this project was focused on exploring themes rather than generalizability (Mcguirk and O'Neill, 2016), 13 interviewees provided enough data for a meaningful analysis and exploration of the topic. All of the interviews lasted at least an hour, with several lasting over 90 min, which provided over 15 h of interviews to transcribe and analyze. The interviews were then analyzed by using a thematic analysis, which allowed me to identify themes in the qualitative data and can be helpful when analyzing data focusing on participants' “experiences” and “understandings and perceptions” (Clarke and Braun, 2016; p. 88). As my project aimed to explore what challenges the cadets felt they may encounter when working in the military, and what influence gender may have, a thematic analysis was well suited for exploring the research question.

Research ethics were taken into consideration at every step of the project, including safely handling the data and anonymizing the participants, and also reflexivity from the researcher (Dowling, 2016). Reflexivity meant that I was paying attention to my own positionality, but also how I interacted and engaged with the data as I was analyzing it. This type of awareness also contributed to ensuring that the research was produced in a rigorous and trusthworthy manner.

## Results

In this section I present interview excerpts to show the main themes that emerged from my thematic analysis. The themes presented include (1) Participants' perceptions that others in the military don't understand cyber; (2) Effectively communicating cyber to non-cyber; and (3) reflections on gendered perceptions of technologies and its impact on female cyberengineers. In the discussion section I relate these themes back to the broader fields of military ethics and gender studies. Pseudonyms are used here to anonymize the identity of participants.

### Participants' perceptions that others in the military don't understand cyber

Several cadets expressed that at a broader level across the military there was a lack of understanding about the role that cyber capabilities play in the military.

Morten: I think many people are not aware of how badly things can go, or how vulnerable systems are. So I don't think that cyberwarriors get enough credit, and often it is understandable, because when you are defending a network, it is not something that everyone physically sees, so it is difficult to understand everything we are doing. Fighter pilots by comparison, it is much easier to acknowledge, and it kind of has more prestige. If you shoot down an enemy aircraft, it is something that you see with the physical eyes, but something in cyber space can be really difficult to understand for normal people.

Others would also state that in addition to a possible lack of understanding, they felt as though some units in the military devalue the importance of cyber. When asked about stereotypes that might exist about cyberengineers in the military, Petter would share he felt cyberengineers were seen as the nerds with less prestige in the military, and how he thought this might impact how others see cyber.

Petter: I will be deployed with field units that have no security professional, other than us cyberengineers, and there is a few of us in each battalion, and there I think we have this nerdy, overly anxious stereotype, that we are the guys who complain that everything they do is unsafe. Sort of a necessary evil. We are sort of the outsiders there. Everyone else is leadership, which is hard work, or you know the guys in infantry, like sharp shooters, and I understand that we can be annoying when we come up and tell them that they don't use their cell phones right.

While Petter shares here that one of the challenges is that other units don't take cybersecurity seriously, and how might stereotypes of cyberengineers as nerds maybe played a role in this, Julia shared that other members of the military are starting to take cyber more seriously.

Julia: I think that people take it more seriously after the attack on Stortinget (Norwegian Parliament) and seeing what an attack can do. But I also think it is misunderstood, because cyber is so broad, and most people think of it as a computer and internet, but it is much more. Communications, satellites, radios, and much more.

Here Julia references a prominent hack that took place against the Norwegian Parliament in 2021 (Stolt-Nielsen and Lysberg, 2021), underscoring that cybersecurity and cyber capabilities can have a significant impact on Norwegian security. However, similar to other comments shown, Julia feels that there is a general misunderstanding of what is the cyber domain. This underscores the need for cyberengineers to be able to communicate what is ongoing in the cyber domain to other members of the military, a theme which many of the cyberengineers themselves pointed out, and which is the next theme I turn to.

### Effectively communicating cyber to non-cyber experts

When asked what skills are needed for cyberengineers, there were a number of responses that emerged, including general technical competence, creativity, and the need to have good communication skills. As Lars shared:

Lars: I think it is important to have a good understanding of ethics. As a cyberengineer you have more understanding of what the technology is, so it is important to be able to communicate to other people in a way so that they can understand.

As Lars highlights, as a cyberengineer not only do you need to be able to communicate in a way that people understand, but also as a cyberengineer you need to be able to explain the ethics associated with the technology (Ellis and Grzegorzewski, 2021) a point returned to in the discussion more fully.

Anne would speak about the importance of good communication skills. Yet when asked if she felt that cyber had prestige in the military, she would share:

Anne: Absolutely not, or not yet at least, and that is something we have talked about quite a bit in our studies from the beginning. We have to dare to speak up, and are likely going to meet resistance, because we are going out as specialists, and not leaders. We have to advise them on something they know nothing about, so it is possibly easier to not consider what we are saying, and our job is trying to describe how what is happening in the cyber domain is important to everything else that is happening.

From an institutional point of view, these comments present important insights into what types of skills and training should be included for cyberengineers, and I return to this in the discussion after presenting the final theme from my analysis.

### Reflections on gendered perceptions of technologies and its impact on female cyberengineers

Gender as a theme would come up first with the women I spoke with when asking the cadets about stereotypes about cyberengineers in the military.

Anne: I think the stereotype is the typical nerd, with glasses and head buried deep in the computer. I think that is still what most people think, and when I tell people I am doing this, they are like, but oh you are a girl, so that is also something that hasn't changed.

Anne was among the first I interviewed, and when I spoke with another of the women in the program, Sara, she also said she didn't feel as though she fit the stereotype of who is a cyberengineer. When I asked if it had anything to do with being a woman she replied:

Sara: Both that (being a woman), and also that I am not a gamer really. I feel like those who are gamers fit the stereotype better.

While both Anne and Sara expressed that they didn't think they fit the stereotype of who is a cyberengineer due to their gender, neither of them felt that being a women had an impact on them being treated differently in the military. However, Julia expressed that she thought there were moments where she was being treated differently because she was a woman.

Julia: Many guys, they don't understand that women also know stuff about computers. And I have experienced it myself during the exercise when we had cyber operations, I had to be really patient, because, they expected less of me than the other guys.

Here we see that Julia feels that because of gendered stereotypes about technology (Corneliussen, 2021), she is seen as less competent when it comes to cyber operations. When speaking with some of the male cadets about gender equality in the military and amongst cyber operators, many of them spoke about the high level of gender equality in the Norwegian military and in cyberengineering. However a few of them did highlight that due to broader societal ideas about technology, this may lead to gendered stereotypes.

Jens: Even me, I don't naturally assume that a woman would be interested in gaming on a PC, so that is a kind of stereotype, that isn't explicitly military, but that is in most of the society.

Interestingly, Jens expresses that it is gendered stereotypes about technological competence, and not about the military that in Norway might create barriers for female cyberengineers. However, as will now be shown in the discussion, gendered dynamics were also at play for the male cyberengineers.

## Discussion

As Jøsok et al. (2017) highlight, cyber applications in the military “distort” military structures, as those lower in ranks often have higher technical competence than their officer (p. 497). My interview findings show that many of the cyberengineers feel challenged by this disruption of hierarchy, and they reflected on how they feel that in the broader military, few understand what the cyber domain is or take it seriously. This further adds to what the cadets feel is a challenge they will encounter when working in the military, which is explaining cyber-related challenges and topics to their commanding officers and fellow soldiers (Knox et al., 2018). This has important implications for military effectiveness, as the cyber domain will increasingly play a vital role, and there will be a need for good communication between “operator and commander... in order to communicate efficiently to support each other's sensemaking” (Jøsok et al., 2017 p. 493). The implementation of AI-enabled systems will add to this challenge of effective communication, as AI further obscures understanding how these technologies work (Ellis and Grzegorzewski, 2021). Yet as Lars comments shows about the ethics of these technologies, he not only feels a burden to communicate the cyber domain, but also to communicate the ethical challenges associated with It. As the use of AI is integrated into cyber operations in the future, it will be crucial that militaries focus on developing the skills to operate and understand AI systems, but also still focus on the development of good communication skills among cyber operators as the personnel will remain crucial despite advanced technologies (Ellis and Grzegorzewski, 2021).

Additionally this article has highlighted the role of gender in relation to effective communication of the cyber domain in the military. As Corneliussen (2021) found with women working in ICT in Norway, “negotiating their belonging” (p. 48) is often more difficult for women than men due to stereotypes about who is good with technology. In an organization such as the military, often seen as a gendered and a masculine institution (Kvarving, 2019), overlapping factors appear to play a role in how these women see themselves as fitting in. While Julia was the only woman who felt as though this had negative consequences, the perception among the women interviewed suggests that they are aware of the gendered institution they exist within. As the Norwegian military currently is made up of about 30% women, it is likely that those who these women would be interacting with, and communicating with about the cyber domain, would likely be men. Experiences of discrimination and perceptions of self which are tied to societal stereotypes can possibly contribute to uncertainty, creating extra barriers in the everyday tasks and assignments these women may work with. The challenge this provides to military institutions is to continue to focus on how they can try to create more gender-neutral perceptions of technology and technological competence, and to take into account how these assumptions may impact female cyberengineers.

Gendered perceptions appear to have an impact on the men as well. None of the men interviewed expressed that they didn't fit the stereotype of who is a cyberengineer, further highlighting the gendered nature of technology and the military and of who feels they fit the stereotype. However, their comments about how they felt cyberengineers were viewed more broadly in the military illustrate their perceptions of masculine hierarchies in the military (Christensen and Kyed, 2022). As Petter shared, he felt that the cyberengineers were seen as overly anxious, reflecting that perhaps they are seen as embodying a “geek masculinity” (Salter, 2018) which is marginalized within the military. Studies on masculine culture in different military contexts have highlighted the way in which different units within the military can construct “hegemonic” ideals of masculinity for their unit, and also how they feel they may be marginalized or looked down upon by other units in the military (Clark, 2018). Based on the comments of Petter and other men I spoke with, they feel that within the broader military cyberengineers are seen as embodying a nerdy masculinity, which for them they feel creates challenges for how seriously they think they will be seen. New technologies being embraced by militaries globally have, and will continue to change the way in which warfare is conducted. From these men it would appear that the cyber domain sits in an arena of tension, one in which it might be looked down up by other units, but one that also will continue to play an increasingly vital role in multi-domain warfare. What is seen as masculine, and thus of value, is fluid, and has changed in the military before. A question that remains is if / when that might happen in the cyber domain in the context of the Norwegian military.

This article has presented initial findings on the types of challenges Norwegian cyberengineers feel they may encounter in the field, and how gender may play a role in this. It is important to acknowledge that these reflections are based upon their own perceptions, and limitations in the project design limit the extent to which this article can claim these women encounter actual biases. Further research is needed to explore the ways in which gendered assumptions and biases may be impacting the male and female cyberengineers during cyber operations. While the research on improving communication skills among those working with the cyber domain is growing, little of this research has taken into consideration gender. As training institutions seek to prepare these cadets for their future role, understanding how these cadets' perceptions and the role of gender in these perceptions, which may align with or differ from reality, can provide important insights for better training and education. These findings also have policy implications, and highlight the need for institutions and organizations to implement gender-sensitive policies that are attentive to local gender dynamics and set concrete goals and measurements for creating more inclusive environments in the military (Millar et al., 2021).

## Data availability statement

The datasets presented in this article are not readily available because data is personal information about participants and is not to be shared. Requests to access the datasets should be directed to kell.jh.fisher@gmail.com.

## Ethics statement

The studies involving human participants were reviewed and approved by Norwegian Centre for Research Data. The patients/participants provided their written informed consent to participate in this study.

## Author contributions

The author confirms being the sole contributor of this work and has approved it for publication.

## Funding

The funding for this project has come from the Research Council of Norway, under the SAMKUL program.

## Conflict of interest

The author declares that the research was conducted in the absence of any commercial or financial relationships that could be construed as a potential conflict of interest.

## Publisher's note

All claims expressed in this article are solely those of the authors and do not necessarily represent those of their affiliated organizations, or those of the publisher, the editors and the reviewers. Any product that may be evaluated in this article, or claim that may be made by its manufacturer, is not guaranteed or endorsed by the publisher.
